# In This Issue

**Published:** 1994

## THE PRIMARY CARE PRACTITIONER’S ROLE

Primary care practitioners routinely encounter the full spectrum of alcohol problems in their patients. Dr. Katharine A. Bradley examines the role of the primary care physician, from diagnosing alcohol problems to treatment and referral. She also provides a method for conceptualizing treatment of these patients by grouping them into categories for receiving primary, secondary, and tertiary care. She shows how these levels of care can be used to prevent and treat the full range of alcohol problems. (pp. 97–104)

## SPECIALIST’S PERSPECTIVE

### The Family Physician

The family physician often has the opportunity to observe the effects that one person’s alcohol problem has on several members of a family. Drs. Kristen L. Barry and Michael F. Fleming discuss how problems with alcohol affect the patient and other family members and suggest ways a family practitioner can help patients and families deal with the problems of alcohol abuse and dependence. (pp. 105–109)

### The General Internist

General internists are well versed in the medical complications of alcohol abuse and alcoholism, which can include destruction of the liver and pancreas as well as deleterious effects on the central and peripheral nervous systems. However, as Dr. Patrick G. O’Connor points out, internists may be less familiar with the physically asymptomatic and early stages of alcohol abuse. He describes the types of alcohol problems internists encounter most often in their practice as well as some methods for diagnosing and managing alcohol abuse and alcoholism. (pp. 110–116)

### The Obstetrician/Gynecologist

Drs. John M. Thorp and Susanne Hiller-Sturmhöfel describe the alcohol-related problems that obstetricians/gynecologists (OB/GYN) encounter most in their practice. The OB/GYN not only must be able to detect and treat the specific medical consequences of alcohol abuse but also to be aware of the social problems and treatment requirements that are unique to women. (pp. 117–120)

### The Pediatrician

Alcohol consumption is illegal for adolescents. Yet by the time they graduate, almost 90 percent of high school students have tried alcohol. Drinking in this younger population may lead to accidents, injuries, or a variety of social or psychological problems. Adolescents also may suffer adverse consequences resulting from their parents’ abuse of alcohol. Drs. Hoover Adger, Jr., and Mark J. Werner review some of the effects of alcohol abuse in adolescents and discuss the role of pediatricians in diagnosing and treating these young patients. (pp. 121–126)

## DETECTING ALCOHOL-RELATED PROBLEMS IN TRAUMA CENTER PATIENTS

Many patients who are admitted to trauma centers have an underlying drug-use problem, most commonly alcoholism. Traditionally, the primary emphasis of trauma center care for alcoholic patients has focused on the treatment of physical injuries. Yet, according to Dr. Carl A. Soderstrom, trauma center clinicians are in a unique position to identify and treat patients with alcohol or other drug problems—treatment that could prevent future injury or loss of life. (pp. 127–130)

## BIOLOGICAL STATE MARKERS OF ALCOHOL ABUSE

Biological markers are among the most promising tools for the early diagnosis and treatment of alcohol-related problems. Dr. Mikko Salaspuro discusses the two types of markers used in clinical practice today. The first type detects recent drinking; the second screens for long-term alcohol abuse. Biological markers also can be used as motivating factors to help patients reduce their drinking. To date, no test can satisfy all the criteria of an ideal marker: sensitivity, specificity, simplicity, and low cost. Potential markers are being evaluated, however, and may eventually come into use. (pp. 131–135)

## EFFECTIVENESS OF TREATMENT

Early detection of alcohol problems is vital if intervention and treatment are to be successful. Dr. David Buchsbaum offers ways in which primary care providers can better detect which of their patients have alcohol problems. He also reviews evidence which shows that even brief (5–15 minute) office-based interventions can positively influence patients’ drinking behavior. (pp. 140–145)

## MEDICAL EDUCATION IN ALCOHOL AND OTHER DRUGS

Biopsychosocial models of disease—which include psychological and social aspects as well as the biomedical elements of a disease or disorder—have found a niche in medical education in recent years. Drs. Catherine E. Dubé and David C. Lewis note that such models are particularly well suited to teaching medical students about alcohol abuse and dependence. The authors also review the evolution of curricula on alcohol and other drug abuse and discuss future trends in substance abuse education. (pp. 146–153)

## THE NEED FOR ALCOHOL ABUSE-RELATED EDUCATION IN NURSING CURRICULA

Nurses have many important and diverse roles in the primary care setting. To be effective in these roles, nurses should, as part of their education, receive clinical training on recognizing and treating patients with alcohol problems. Dr. Madeline A. Naegle describes both the history and the current state of nursing curricula as it relates to the topic of substance abuse. She also reviews model curricula programs now in existence and discusses future developments in the teaching field. (pp. 154–157)

## THE ROLE OF NURSES

Nurses are frequently the first contact patients with alcohol problems have with the health care system. Drs. Eleanor J. Sullivan, Sandra M. Handley, and Helen Connors discuss the unique role that nurses play in the primary care setting. They review the characteristics that make nurses especially qualified to care for patients with alcohol problems as well as those that may interfere with the nurse’s ability to deliver appropriate care. (pp. 158–161)

## PREVALENCE OF PHYSICIAN SCREENING FOR ALCOHOL USE

Patients who visit primary care physicians may not recognize that drinking is the source of their medical problem. Physicians, through screening for alcohol problems, must be able to make this connection if they are to prevent and/or treat the medical consequences of alcohol abuse. In this Epidemiologic Bulletin, Dr. Diane Deitz and her colleagues review a survey in which primary care patients were asked whether physicians screened for alcohol consumption during routine office visits. They discuss how these results differed among demographic groups and among patients with various levels of alcohol consumption. (pp. 162–168)

